# Metastatic breast cancer: the potential of miRNA for diagnosis and treatment monitoring

**DOI:** 10.1007/s10555-015-9551-7

**Published:** 2015-02-27

**Authors:** Andrew McGuire, James A. L. Brown, Michael J. Kerin

**Affiliations:** Discipline of Surgery, School of Medicine, National University of Ireland, Galway, Ireland

**Keywords:** miRNA, Breast cancer, Metastatic, Biomarker, Metastatic sites, Metastatic rates, Subtype

## Abstract

Breast cancer affects approximately 12 % women worldwide and results in 14 % of all cancer-related fatalities. Breast cancer is commonly categorized into one of four main subtypes (luminal A, luminal B, human epidermal growth factor receptor 2 (HER2) positive and basal), indicating molecular characteristics and informing treatment regimes. The most severe form of breast cancer is metastasis, when the tumour spreads from the breast tissue to other parts of the body. Significantly, the primary tumour subtype affects rates and sites of metastasis. Currently, up to 5 % of patients present with incurable metastasis, with an additional 10–15 % of patients going on to develop metastasis within 3 years of diagnosis. MicroRNAs (miRNAs) are short 21–25 long nucleotides that have been shown to significantly affect gene expression. Currently, >2000 miRNAs have been identified and significantly, specific miRNAs have been found associated with diseases states. Importantly, miRNAs are found circulating in the blood, presenting an opportunity to use these circulating disease-related miRNAs as biomarkers. Clearly, the identification of circulating miRNA specific to metastatic breast cancer presents a unique opportunity for early disease identification and for monitoring disease burden. Currently however, few groups have identified miRNA associated with metastatic breast cancer. Here, we review the literature surrounding the identification of metastatic miRNA in breast cancer patients, highlighting key areas where miRNA biomarker discovery could be beneficial, identifying key concepts, recognizing critical areas requiring further research and discussing potential problems.

## Introduction

**Fig. 1:**
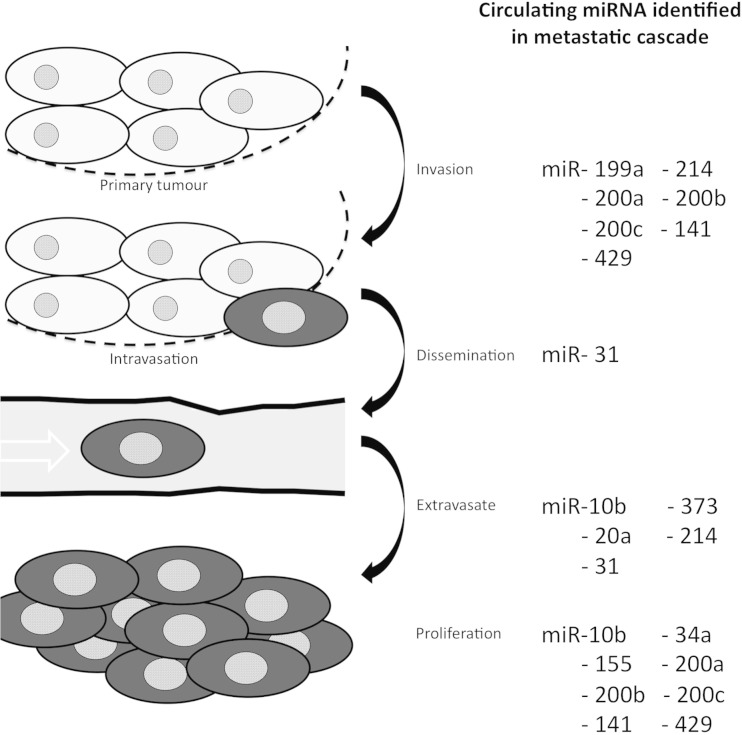
Stages of breast cancer metastasis. *Left*: Order of process resulting in breast cancer metastasis. *Right*: Circulating miRNA associated with key steps in the metastatic cascade

Breast cancer is the second most common cancer diagnosed worldwide, affecting approximately one in eight women during their lifetime [1]. It affects 1.3 million women each year and accounts for 23 % of all cancer cases and 14 % (465,000) of all cancer-related deaths [2]. The most severe form of breast cancer occurs when the cancer spreads from the breast tissue to other regions of the body (metastasis), significantly increasing the tumour burden and often resulting in a fatal diagnosis. Breast cancer metastasis follows a cascade starting with local invasion of the surrounding tissue, spreading into the blood or lymphatic vessels and ending with dissemination of tumour cells to distal organs [3, 4] (Fig. 1, left). Despite modern treatments, metastatic breast cancer (MBC) is often incurable, with up to 5 % of patients presenting with distal metastases at time of diagnosis [2]. Currently, distal metastasis (M1) occurs in 10–15 % of patients within the first 3 years. Furthermore, approximately one third of women who have breast cancer with no lymph node involvement at time of diagnosis will develop distal metastases [5]. Significantly, the rate and site of metastasis can vary largely and is thought to be dependent on primary tumour subtype. Clearly, further knowledge is needed to both diagnose and treat metastatic breast cancer. Recently, microRNAs (miRNAs) have shown promise as new biomarkers for many cancers, including metastatic breast cancer [6–8]. Importantly, miRNAs have been linked to all stages along the metastatic cascade in breast cancer [9–15] (Fig. 1, right). Here we examine studies using circulating miRNAs as biomarkers for metastases, markers for tumour recurrence and response to clinical treatments. We likewise discuss the potential applications of miRNA for therapeutic metastatic breast cancer diagnosis, treatment and basic research.

## MicroRNA

**Fig. 2:**
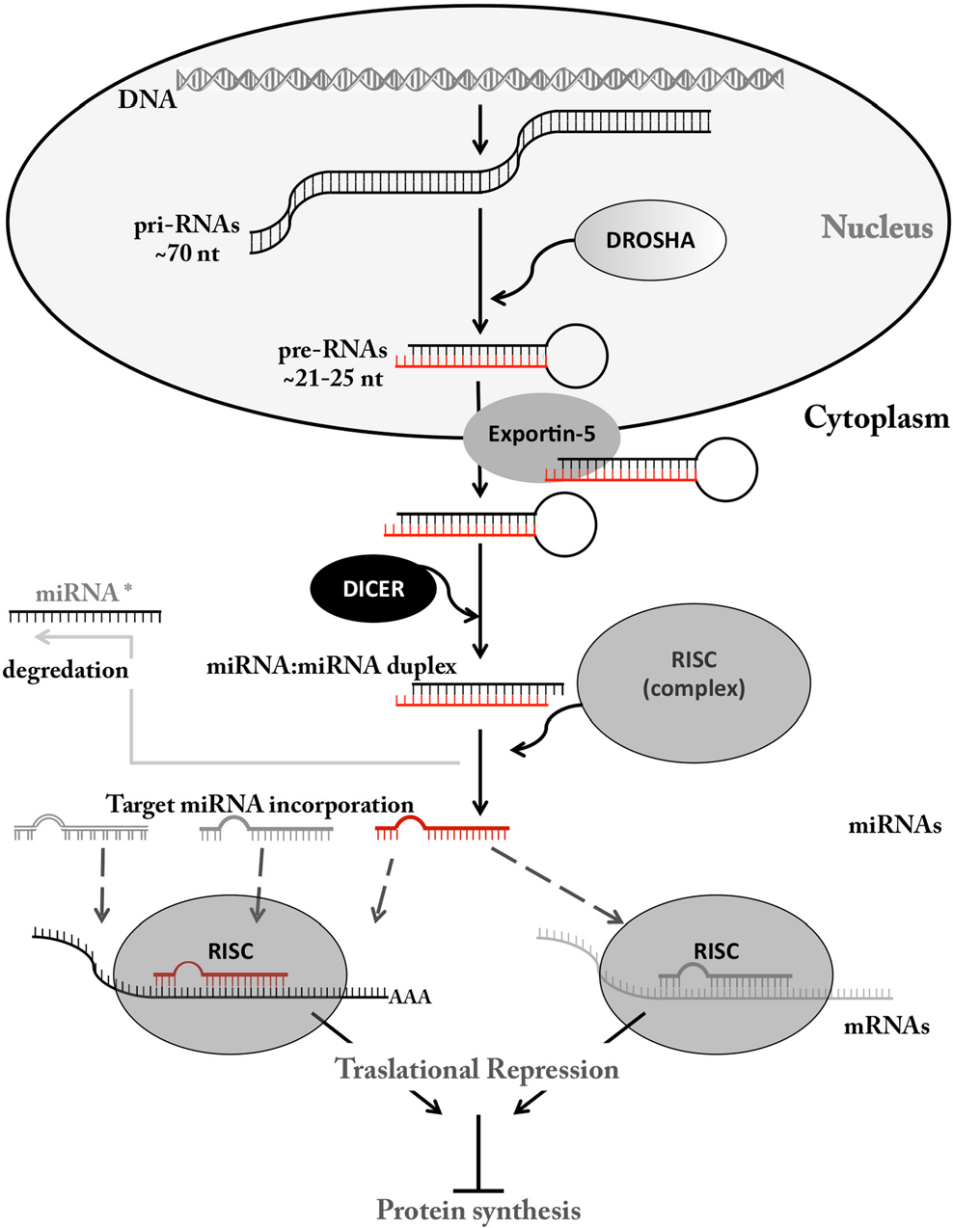
miRNA biogenesis and mechanism of action

MicroRNAs (or miRNAs) were originally discovered in the early nineties in Caenorhabditis Elegans [16]. MicroRNAs are a 21–25 long class of small non-protein coding RNA that function as gene regulators by inhibiting the degradation of their target mRNAs and inhibiting translation (Fig. 2). miRNAs have been demonstrated to be involved in cell development, differentiation, proliferation and apoptosis [16]. The first human, disease-related miRNA characterized was from chronic lymphocytic leukemia [17] and subsequently, circulating miRNA were identified in patients with diffuse large B-cell lymphoma [18]. Consequently, miRNAs were linked to many other diseases and cancers [19, 20]. Since then, >2000 miRNAs have been identified in humans and these miRNAs regulate an estimated 30 % of all human genes [21]. miRNA can exert their action in cancers through both tumour suppression and oncogenic mechanisms (as oncomirs) [16, 22]. Fragile sites and genomic regions involved in oncogenic rearrangements in cancer are similarly thought to influence the production of cancer-related miRNA [23]. Furthermore, as a proof-of-principle for any potential therapeutic application of miRNA, circulating miRNAs have been identified which correlate with breast cancer subtypes (Table 1) [24–27].

**Table 1:** Breast cancer molecular subtypes

Breast cancer subtype	Molecular subtypes			Subtype-specific circulating miRNA	Ref
	ER	PR	HER2		
Luminal A	+	+	−	miR-29a, miR-181a, miR-652	[24]
Luminal B	+	+	+	miR-342	[25]
HER2 + ve	+	−	+	miR-10b, miR-21	[26]
Basal (TNBC)	−	−	−	miR-210	[27]

### miRNA biogenesis and action

miRNAs are formed from precursors called pri-miRNAs that are processed in the nucleus by Drosha, an RNA III type nuclease. These pri-miRNAs are transported to the cytoplasm by exportin-5, where they are cleaved by Dicer, another RNAse III enzyme, forming an asymmetric duplex (miRNA:miRNA). This miRNA duplex is then separated, and the mature target miRNA molecule is incorporated into the RNA-induced silenced complex (RISC) where it binds a member of the Argonaute (Ago) protein family [the other miRNA molecule (miRNA*) is normally degraded] [16, 28, 29]. The active RISC complex is then able to target mRNA transcripts with a sequence complementary to the mature incorporated miRNA molecule, leading to inhibition of protein expression (Fig. 2). miRNA can be exported from cells packaged in membrane-bound extracellular compartments (exosomes) or bound to RNA binding proteins [30]. Exosomes provide another method of extracellular signalling as they are able to bind and merge with other cells, thus influencing their environment. Furthermore, exosomes have been directly implicated in cancer [31]. Significantly, miRNAs are differentially secreted or selectively packaged into exosomes, with different cell and tumour types displaying distinctive miRNA profiles [32].

### Breast cancer diagnosis

Currently, breast cancer can be subcategorized based on the status (+/−) of the hormone receptors oestrogen receptor (ER) and progesterone receptor (PR) and the Receptor tyrosine-protein kinase erbB-2 (ERBB2 or HER2). Furthermore, recent genetic testing has enabled the molecular subtyping of breast cancers [33, 34]. Presently, there are four major molecular subtypes: luminal A, ~50–60 % of breast cancers; luminal B, 10–20 %; HER2 + ve, 15–20 %, with the remaining 10–20 % considered Basal subtype [35]. Further subcategorizing the common molecular breast cancer subtypes has allowed clinicians to tailor treatments to each individual patients cancer [36]. In particular, the Oncotype DX test evaluates 16 cancer-related genes and 5 reference genes, with the results used to estimate the likely reoccurrence in patients and diagnostically to determine if a patient should receive chemotherapy [37]. Significantly, as miRNAs have been implicated in cancer metastasis, miRNA signatures are being pursued as novel clinical diagnostic targets to allow further subtyping of breast cancer and for predicting metastasis or therapeutic resistance [38–41]. The potential of miRNA as biomarker targets is facilitated by their stability in blood and their ability to withstand repeated freezing and thawing cycles [42].

### miRNA in metastatic breast cancer tissue

Identifying and categorizing miRNAs expressed during the different stages of metastases will accelerate any therapeutic potential of these biomarkers, while illuminating the underlying mechanisms of cancer [43]. Currently, a number of studies have investigated miRNA expression profiles (upregulation or downregulation) of metastatic breast cancer tissue, providing insights into the processes of breast cancer initiation, progression and maintenance [44–47]. Significantly, it has been demonstrated that restoring the expression of individual miRNA observed to be lost in breast cancer models (such as miR-31, miR-126 or miR-335) can suppress metastasizes *in vivo* [48, 49]. Additionally, it has been suggested that cancer stem cells may influence metastasis [50–52], which would further contribute to any breast cancer miRNA profile. It is hoped that identifying breast cancer-specific miRNA and their functional relevance will lead to improvements in the early detection and treatment of tumours, particularly in younger patients.

### Metastases

**Fig. 3:**
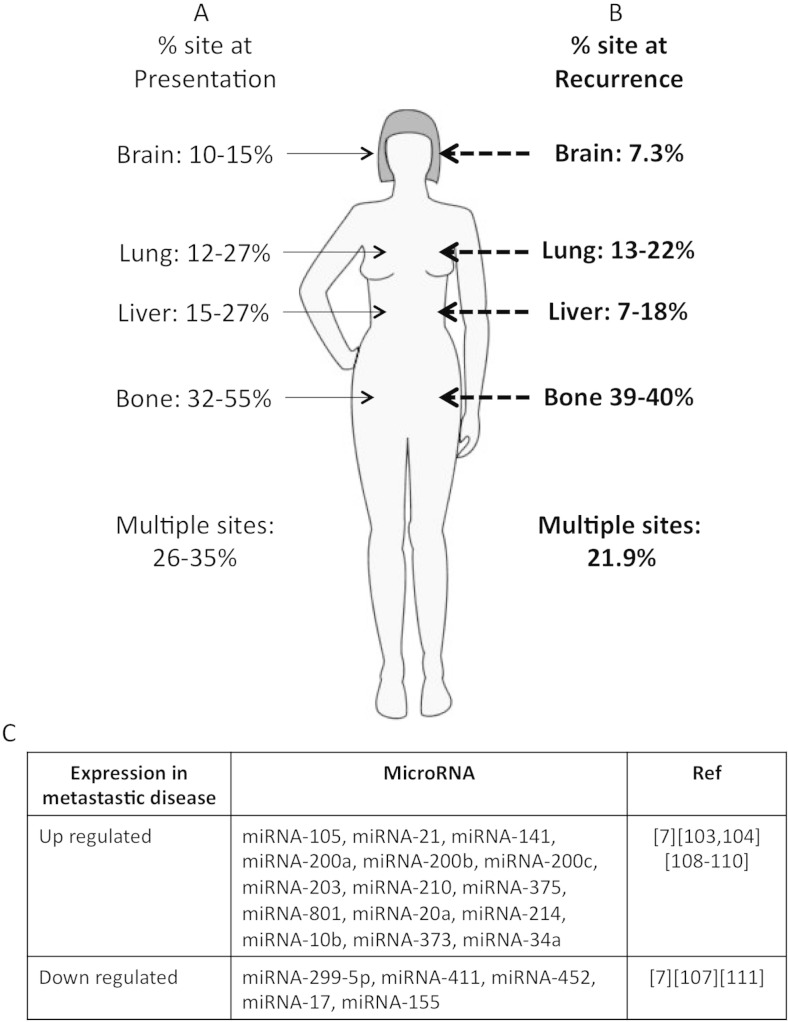
**a** Sites of metastatic breast cancer at presentation/diagnosis. **b** Recurrence sites of metastatic breast cancer. **c** Summary of published miRNA-associated breast cancer

Breast cancer metastasises though the lymphatic system or via the circulatory system and is the overwhelming cause of mortality in patients with malignancies, causing 90 % of deaths in solid tumours [53]. Metastasis in breast cancer is characterized by a distinctive spread via regional lymph nodes to the lungs, liver, brain and bones [54]. Importantly, the rates and sites of distal metastasis can vary depending on age and stage of diagnosis [55, 56]. The most common site of metastases is the bone, often the first site of distal metastases in up to 50 % of patients [57], with lungs and liver as the second and third most common metastatic sites (respectively) (Fig. 3a, left). Significantly, 10–15 % of metastatic breast cancer patients will develop brain metastases, making breast cancer the second most common source of brain metastasis [58]. A similar distribution of metastasis is seen following relapse (post-treatment), with ~22 % of patients having multiple sites of metastasis (Fig. 3b, right) [59, 60].

Further examining metastasis by molecular breast cancer subtypes, distinctive patterns of metastasis sites are observed (Table 2). Bone metastases remain the most common metastatic site in luminal A, B and HER2 + ve breast cancers [59, 61, 62]. However, basal cancers were found to primarily metastasize to the lungs [63]. Interestingly, luminal cancers also tend to have a lower rate of brain metastases. Significantly, brain metastasis for the HER2 + ve subtype is high [64], despite the implementation of trastuzumab-based treatments for HER2 + ve breast cancers in the late 1990s. As Herceptin is not expected to cross the blood brain barrier, it is not believed to have influenced these rates. Currently, a number of circulating miRNA have been identified that are dysregulated (up or down regulated) in breast cancer metastasis (Fig. 3c). Identifying circulating miRNA associated with distinct metastatic sites could provide another powerful diagnostic tool for clinicians to evaluate disease stage and monitor progression.

**Table 2:** Sites of breast cancer metastasis by molecular subtypes

Metastasis sites	Breast cancer subtype			
	Luminal A	Luminal B	HER2 + ve	Basal
Brain	6.6 %	8.2 %	23.3 %	18.1 %
Lungs	25.1 %	29.2 %	32.4 %	35.4 %
Bone	62.1 %	64.5 %	47.7 %	32.2 %
Liver	25.1 %	26 %	39.9 %	23.8 %

### Subtype and metastasis stage

Currently, the severity of a person’s breast cancer is based on the TNM staging, where T describes the tumour size, N defines the lymph node status (+/−) and M relates to any distant metastases (0/1). In addition to TNM staging, breast cancer can be also divided into groups, stages I–V, depending on size and metastatic spread. Significantly, this combined staging is used clinically to inform the choice of treatment regime. However, TNM staging has its limitations and drawbacks, including overtesting and uncertainties in staging due to limits in sampling auxiliary lymph nodes. Furthermore, the four breast cancer molecular subtypes contain different disease progression, survival and relapse rates. Luminal subtypes tending to have slower metastatic spread, lower reoccurrence rates and better outcomes than HER2 + ve or basal subtypes [61, 65–69]. This difference is independent of histological subtype or time of detection, with the majority of basal carcinomas detected in the early stages of breast cancer. When comparing median survival (from time of first distal metastasis), luminal A and B subtypes display longer overall survival (2.2 and 1.6 years) compared to HER2 + ve subtype (1.3 years). However, the basal subtype has the worst overall survival rate (0.7 years). This is reflected in the presentation rates of the metastatic disease, stratified by molecular subtype (Table 3).

**Table 3:** Metastasis of breast cancer molecular subtypes (approximates)

Breast cancer subtype	% Metastasis at presentation	% Metastasis at recurrence	MBC median survival (years)
Luminal A	2–2.6	27.8	2.2
Luminal B	1–2.5	42.9	1.6
HER2	5–6	51.4	1.3
Basal	4–5	35.1	0.7

Significantly, the relapse rates vary considerably by subtype, with HER2 + ve the highest (51.4 %), followed by luminal B (42.9 %), basal (35.1 %) and luminal A (27.8 %). Interestingly, in addition to the lowest recurrence rate, luminal A relapse also occurs later than the other subtypes [61]. Clinically, HER2 + ve cancers have a poor prognosis; however, following development of anti-HER2 treatments, there has been an improvement in disease-free survival (from 72.2 to 78.6 %) [70, 71]. Bone remains the most common primary metastatic site, while luminal B has a higher rate of metastases to other visceral organs (such as liver), compared to luminal A. The basal subtype often presents with a younger onset, larger mean tumour size and higher grade, with the lowest overall survival [72, 73].

MicroRNAs have the potential to provide an additional mechanism for classifying breast cancer subtypes and tracking disease progression. A number of studies have investigated microRNAs in tissue as a means for identifying the main molecular breast cancer subtypes [24–26, 74–76]. Importantly, recent studies have found that microRNAs (miR-210, miR-328, miR-484 and miR-874) have the potential to predict prognosis or risk of recurrence [26, 77, 78]. Furthermore, it has been shown that microRNAs may be able to identify a subtype-specific response to treatment [27, 79, 80].

### Breast cancer treatments

Currently, surgery is the primary treatment for early stage breast cancer. However, the use of chemotherapy, radiotherapy and hormone therapy has vastly improved survival rates [81–83]. For the treatment of metastatic breast cancer, chemotherapy and radiotherapy are used in the neoadjuvant setting, before breast conservative surgery or mastectomy and axillary node clearance. In progressive disease (stage IV), chemotherapy and radiotherapy are the principal treatments, along with hormone therapy. Defining metastatic specific miRNA has the potential to categorize breast cancer and inform and improve treatment choices. Furthermore, the use of specific miRNA as therapeutics has the potential to one day become a valid treatment option [84–86]. Indeed, there are current clinical trials investigating the efficacy of using miRNA to treat cancer [87].

### Chemotherapy and miRNA

Chemotherapy usually involves a combination of drugs and is the leading treatment, often combined with hormone therapy, in metastatic breast cancer. The most common chemotherapeutics used are anthracyclines (doxorubicin and epirubicin), taxanes (paclitaxel and docetaxel), fluorouracil (5-FU) and cyclophosphamide. Currently, there is no evidence of benefit of one regime over another. However, a meta-analysis has indicated a benefit of adding taxanes to an anthracycline-based regime, demonstrating a 5 year risk reduction of 5 % in disease-free survival and 3 % in overall survival [88]. Significantly, HER2 + ve patients treated with trastuzumab in combination with chemotherapy had increased median survival rates from 20.3 to 25.1 months [89]. Despite these treatment advancements, a large proportion of patients do not respond to traditional chemotherapy or hormone therapy [90]. In this context, circulating miRNAs have been explored as potential biomarkers, to predict treatment response [91–96]. Currently, only a few recent studies have explored the relationship of miRNAs with subtype-specific treatment [94, 97, 98] (Table 4). The early identification of circulating miRNA that can diagnose disease and/or chemotherapeutic responses will greatly facilitate improved treatments, leading to better outcomes for patients.

**Table 4:** Metastatic breast cancer treatment regimes by subtype

Subtype	Circulating miRNA	Menopause	Node negative	Node positive
Luminal A	miR-19a, miR-205	Pre	Tamoxifen ± chemotherapy	Chemotherapy + tamoxifen ± ovarian ablation
		Post	Aromatase inhibitor (AI) + tamoxifen ± chemotherapy	Chemotherapy + AI with tamoxifen
Luminal B	N.D	Pre	Tamoxifen + Herceptin ± chemotherapy	Chemotherapy + Herceptin + tamoxifen
		Post	AI with tamoxifen + herceptin ± chemotherapy	Chemotherapy + herceptin + AI with tamoxifen
HER2	miR-210	Pre	Herceptin + chemotherapy	Herceptin + chemotherapy
		Post	Herceptin + chemotherapy	Herceptin + chemotherapy
Basal	miR-27a, miR-30e, miR-155, miR-493	Pre	±Chemotherapy	Chemotherapy
		Post	±Chemotherapy	Chemotherapy

Bone disease adds denosumab, zoledronic acid or pamidronate to chemotherapy regime

## Diagnosing metastatic disease

Mammography is the gold standard for breast cancer screening, but it is mainly used for detection of local disease and is unreliable for diagnosing metastatic disease, with a false positive rate of ~50 % (7–9 % of these patients require a biopsy) [99]. Sentinel lymph nodes are the first lymph node in a tumour bed that receives lymphatic drainage from the tumour tissues, and sentinel lymph node biopsy (SLNB) currently provides the most accurate diagnosis for metastatic disease [100]. Currently, SLNB is recommended for early breast cancer, without any clinical evidence of nodal involvement [101]. However, SLNB only diagnoses regional metastasis. If distal metastases are suspected, SLNB needs to be combined with additional techniques, such as imaging. The development of an accurate biomarker, such as circulating miRNA, to diagnose or predict metastatic spread, could negate/reduce the need for many patients to undergo invasive procedures or surgery.

### Circulating miRNA as biomarkers in metastatic breast cancer

Identifying circulating miRNA to use as biomarkers for metastatic breast cancer is currently a key priority for many research groups (Table 5). The first miRNA shown to be highly expressed in metastatic breast cancer was miR-10b (using mouse and human cells), with a clinical correlation in primary breast carcinomas [111]. A subsequent study confirmed this, finding elevated miR-10b, miR-34a and miR-155 levels in patients with metastatic breast cancer [7]. Further supporting this, it was recently shown that miR-10b and miR-373 were increased in lymph node positive breast cancer [108]. Excitingly, a significant increase in circulating miR-10b and miR-373 was demonstrated in lymph node positive patients, compared to patients with no nodal involvement or healthy controls. Differences in miRNA levels in lymph node positive patients were also observed in a subsequent study [106], where higher levels of miR-20a and miR-214 were found in lymph node positive patients, compared to lymph node negative patients. miR-210 was also identified as a potential marker for lymph node metastasis, however only in a small cohort [94]. Interestingly, miR-10b was identified as a potential biomarker for brain [109] and bone [110] metastases in breast cancer. Together however, these independent results cast doubt on the use of miR-10b as a metastatic specific marker. Furthermore, miR-299-5p and miR-411 were found to have significant differences in metastatic breast cancer patients, with the additional miRs miR-215 and miR-452 of interest, without reaching statistical significance [105]. Additionally, miR-21 has also been identified as a marker for breast cancer and predictor of stage [103]. Recently, eight miRNAs (miR-141, miR-200a, miR-200b, miR-200c, miR-203, miR-210, miR-375 and miR-801) were found to be significantly higher in patients with circulating tumour cells (CTC) [104]. In another study, higher levels of miR-105 were found in early onset breast cancers that metastasized, compared to cancer that did not [102]. This study also found that overexpression of miR-105 promoted metastasis *in vivo*. The miRNAs miR-17 and miR-155 have been identified as potential differentiators between metastatic and non-metastatic breast cancer [107]. Supporting those studies, a number of these miRNAs have previously been identified as markers in metastatic triple negative breast cancer samples [79]. As the above publications did not specifically state the sites of metastases, this may partially account for the diverse miRNA identified. Importantly, these studies highlight the importance and potential application of circulating miRNA as biomarkers that can discriminate non-metastatic from metastatic breast cancer.

**Table 5:** MicroRNA in metastatic tumours

MicroRNA	Tumour	Ref.	Cohort (*N*)
miRNA-105	Breast (serum)	[102]	38 Patients
miRNA-21	Breast (serum)	[103]	102 Patients, 20 controls
miRNA-141, miRNA-200a, miRNA-200b, miRNA-200c, miRNA-203, miRNA-210, miRNA-375, miRNA-801	Breast (serum)	[104]	61 Patients, 76 controls
miRNA-215, miRNA-299-5p, miRNA-411, miRNA-452	Breast (serum)	[105]	75 Patients, 20 controls
miRNA-20a, miRNA-214	Breast (serum)	[106]	48 Patients, 54 controls
miRNA-210	Breast (serum)	[94]	8 Patients, 31 controls
miRNA-17, miRNA-155	Breast (serum)	[107]	72 Patients, 40 controls
miRNA-10b, miRNA-373	Breast (serum)	[108]	35 Patients, 10 controls
miRNA-10b, miRNA-34a, miRNA-155	Breast (serum)	[7]	30 Patients, 29 controls
miRNA-10b	Breast (serum)	[109]	20 Patients, 10 controls
miRNA-10b	Breast (serum)	[110]	122 Patients, 59 controls
miRNA-10b	Breast (serum)	[111]	23 Patients
miRNA-126, miRNA-335	Breast (tissue)	[48]	11 Patients
miRNA-21, miRNA-139-5p, miRNA-486-5p	Breast (tissue)	[9]	6 Patients
Let 7i, miRNA-16, miRNA-26a, miRNA-27a, miRNA-143, miRNA-196a, miRNA-375, miRNA-503, miRNA-519a, miRNA-519b-3q, miRNA-361-5p	Breast (tissue)	[10]	48 Patients
miRNA-27b-3q, miRNA-107, miRNA-103a-3p	Breast (tissue)	[11]	58 Patients
miRNA-22	Breast (tissue)	[112]	108 Patients
miRNA-373	Breast (tissue)	[113]	11 Patients
miRNA-21	Breast (tissue)	[114]	113 Patients
miRNA-15a, miRNA-103, miRNA-148a, miRNA-320a, miRNA-451, miRNA-596	Colon (serum)	[115]	30 Patients
miRNA-27b, miRNA-158a, miRNA-326	Colon (serum)	[116]	150 Patients
miRNA-29a	Colon (serum)	[117]	20 Patients
miRNA-200c	Colon (serum)	[118]	182 Patients
miRNA-141	Prostate (serum)	[119]	21 Patients
miRNA-141	Prostate (serum)	[120]	56 Patients
miRNA-141, miRNA-375, miRNA-378	Prostate (serum)	[121]	84 Patients
miRNA-20a, miRNA-203	Cervical (serum)	[122]	80 Patients
miRNA-21, miRNA-27a, miRNA-106b, miRNA-146a, miRNA-148a, miRNA-223	Gastric (serum)	[123]	20 Controls, 16 patients

Additionally, miRNAs have been identified in metastatic breast cancer tissue samples [9–11, 48, 112–114]. While there is some correlation between circulating and tissue microRNAs, again a large diversity of miRNAs were identified. The number of individual non-overlapping miRNAs identified highlights the complexity of metastasis, staging and breast cancer subtype definition. This emphasizes the need for further comprehensive investigations using similar comparable experimental methodology with more defined/improved breast cancer typing criteria.

## Circulating miRNA in other metastatic tumours

While the identification of circulating miRNA in breast cancer is progressing rapidly, exploring miRNAs investigated in other (often related) metastatic cancers may inform current work in the breast cancer field. Significantly, many of the circulating miRNA observed in other metastatic diseases have likewise been observed in metastatic breast cancer studies.

### miRNA in metastatic colon cancer

In metastatic colon cancer, a significant increase in miR-29a in early liver metastasis was found [117]. In addition, high serum levels of miR-200b showed significant correlation with lymph node and distal metastatic disease in colorectal cancers [118]. Interestingly, miR-200b was identified as an independent predictor of tumour reoccurrence in colon cancer.

Furthermore, tumour reoccurrence in colon cancer was found to be predicted by a panel of six miRNAs (miR-15a, miR-103, miR-148a, miR-320a, miR-451 and miR-596) [115]. Recently in metastatic colon cancer, three miRNAs (miR-106a, miR-130b and miR-484) were found to be significantly overexpressed in patients not responding to first-line chemotherapy [116].

### miRNA in metastatic cervical cancer

In cervical squamous cell carcinoma, a group of six miRNAs (miR-20a, miR-1246, miR-2392, miR-3147, miR-3162-5p, miR-4484) were found to identify lymph node metastasis [124]. Supporting this, miR-20a was found to be significantly increased in patients with lymph node positive cervical cancer, compared to both controls and patients with lymph node negative disease [122].

### miRNA in metastatic gastric cancer

A recent study identified six miRNAs significantly increased in lymph node metastases of gastric cancer: miR-21, miR-27a, miR-106b, miR-146a, miR-148a and miR-223 [123]. In addition, a significant increase in levels of miR-21, miR-146a and miR-148a were found to correlate with increased spread in the lymph node.

### miRNA in metastatic prostate cancer

Recent work in metastatic prostate cancer found miR-141 to accurately predict treatment response, compared to standard markers such as prostate-specific antigen (PSA), lactate dehydrogenase and circulating tumour cells [119]. Interestingly, levels of miRA-141 were found to be elevated in bone metastatic prostate cancer [120]. Importantly, miR-141 expression levels were found to correlate to alkaline phosphatase but not to PSA. A further study looking at metastatic castration resistant prostate cancer again found miR-141, plus miR-375 and miR-378 to be overexpressed compared to low-risk localized patients [121].

## Conclusions

Our understanding of metastatic breast cancer has advanced considerably over the last number of years, yet metastasis remains the major cause of morbidity and mortality in breast cancer. Up to one in three breast cancer patients diagnosed will develop metastatic breast cancer, and despite current treatments, 78 % of these will die within 5 years. Clearly, there is an urgent need to find new clinically relevant biomarkers and tests to allow the early detection of metastatic breast cancer and for the monitoring of treatment response.

The defining of different molecular breast cancer subtypes has significantly aided the treatment of breast cancer, allowing more tailored individual treatment regimes. Significantly, treatment using hormone therapies and Herceptin has increased survival in luminal and HER2 positive breast cancers. However, basal (triple negative) breast cancers continue to have poorer outcomes. Chemotherapy remains the major treatment for metastatic breast cancer, yet similar regimes are given for all subtypes. Additional research is needed to define further subtypes and identify new markers that predict their response to chemotherapy. MicroRNAs have emerged as one such potential marker for predicting metastatic disease and response to treatment. Recently, a small number of miRNAs have shown increased expression in the circulation of metastatic breast cancer patients. In particular, miR-10b has been highlighted across five studies and has been linked to specific sites of distal metastasis. Despite the differences in the rate of metastasis across the breast cancer subtypes, to our knowledge, only one study (Dai et al.) has assessed the miRNA profiles associated with each molecular subtype [125]. Importantly, this study used tumour samples, not circulating miRNA, and did not include metastatic disease. A study investigating circulating miRNA profiles in patients with different molecular subtypes and metastatic disease is greatly needed. Furthermore, the use of different extraction methods and starting material may explain the lack of consensus between microRNAs currently identified in the indicated studies. However, another possible reason may be the diversity of sites of distal metastasis in each study. Only two studies have explored metastatic site-specific miRNAs, Ahmad et al. [109] (brain) and Zhao et al. [110] (bone), with both studies identifying elevated miR-10b. The identification of truly site- or subtype-specific metastatic miRNA may provide the diagnostic tool required to improve personalized metastatic breast cancer treatments. Interestingly, many circulating miRNAs identified in metastatic breast cancer were also found in studies of other metastatic cancers. This may indicate that the identified miRNAs are indicative of the sites of metastasis or that the miRNAs correspond to common underlying mechanisms of cancer metastasis.

Here, we highlighted the current knowledge and potential of microRNAs as biomarkers for improving the diagnosis and treatment of metastatic breast cancer. However, significant further directed research is needed to identify and confirm miRNA that can predict site-specific metastasis disease outcome or patient response to treatments.
